# Guidelines for guideline developers: a systematic review of grading systems for medical tests

**DOI:** 10.1186/1748-5908-8-78

**Published:** 2013-07-10

**Authors:** Gowri Gopalakrishna, Miranda W Langendam, Rob JPM Scholten, Patrick MM Bossuyt, Mariska MG Leeflang

**Affiliations:** 1 Department of Clinical Epidemiology, Biostatistics and Bioinformatics, Academic Medical Center, University of Amsterdam, P.O Box 22700, Amsterdam, DE, 1100, The Netherlands; 2 Dutch Cochrane Centre. Academic Medical Center J1b-226, Postbus 22660, Amsterdam, DE, 1100, The Netherlands

**Keywords:** Grading systems, Quality of evidence, Diagnostic, Medical tests, Grade, Guideline development

## Abstract

**Background:**

A variety of systems have been developed to grade evidence and develop recommendations based on the available evidence. However, development of guidelines for medical tests is especially challenging given the typical indirectness of the evidence; direct evidence of the effects of testing on patient important outcomes is usually absent. We compared grading systems for medical tests on how they use evidence in guideline development.

**Methods:**

We used a systematic strategy to look for grading systems specific to medical tests in PubMed, professional guideline websites, via personal correspondence, and handsearching back references of key articles. Using the Appraisal of Guidelines for Research and Evaluation (AGREE) instrument as a starting point, we defined two sets of characteristics to describe these systems: methodological and process ones. Methodological characteristics are features relating to how evidence is gathered, appraised, and used in recommendations. Process characteristics are those relating to the guideline development process. Data were extracted in duplicate and differences resolved through discussion.

**Results:**

Twelve grading systems could be included. All varied in the degree to which methodological and process characteristics were addressed. Having a clinical scenario, identifying the care pathway and/or developing an analytical framework, having explicit criteria for appraising and linking indirect evidence, and having explicit methodologies for translating evidence into recommendations were least frequently addressed. Five systems at most addressed these, to varying degrees of explicitness and completeness. Process wise, features most frequently addressed included involvement of relevant professional groups (8/12), external peer review of completed guidelines (9/12), and recommendations on methods for dissemination (8/12). Characteristics least often addressed were whether the system was piloted (3/12) and funder information (3/12).

**Conclusions:**

Five systems for grading evidence about medical tests in guideline development addressed to differing degrees of explicitness the need for and appraisal of different bodies of evidence, the linking of such evidence, and its translation into recommendations. At present, no one system addressed the full complexity of gathering, assessing and linking different bodies of evidence.

## Background

Guideline panels aim to develop recommendations based on the best available evidence. In this process, panels often grade the quality of the available evidence [1]. Such an approach of systematically evaluating the evidence and developing recommendations can reduce bias and provide transparency to the guideline development process with benefits to both guideline developers and their target audiences [1]. This has led to the development of a number of systems to grade evidence according to its quality and the strength of recommendations. A review of grading systems in 2002 reported more than 60 available systems existing with wide variations in quality [2].

For intervention studies, the highest available evidence comes from high quality systematic reviews of randomized controlled trials that document the effect on patient important outcomes, such as mortality, morbidity, or functional health [3,4]. Guideline developers making recommendations on medical tests face a particularly unique challenge of developing recommendations that take into account patient important outcomes, because such direct evidence of the effects of testing on outcomes often does not exist. The available evidence base is often in the form of studies of the analytical performance and the reproducibility of medical tests, and diagnostic accuracy studies that estimate the sensitivity and specificity of a test in comparison to the clinical reference standard for a disease [5]. Yet, diagnostic test accuracy is not always appropriate for expressing test performance. Guideline panels may be interested in producing guidelines to address the use of tests for testing disease susceptibility and risk stratification, for staging a disease, monitoring its course for treatment selection, and for surveillance after treatment. In these applications, diagnostic test accuracy data may not always be the most informative expression of test performance [6,7].

Hence, when developing recommendations for a medical test, guideline developers need to go beyond the analytical performance and the accuracy of a test to consider consequences of testing on patient outcomes, and to balance benefits versus harms of testing, taking into account patient values and preferences and resource implications [8].

In addition, tests are never administered in isolation but as part of a testing strategy. Guideline panels therefore need to consider the evidence in context of the most likely testing strategy that includes the test that is being evaluated, and from there make judgments on the downstream patient consequences for further testing and treatment [9]. All of this implies that guideline panels need to use an evidence-grading system that allows for the assessment of the quality and linkage of different types of indirect evidence in as transparent and structured a manner as possible.

Grading systems generally describe a subset of steps that are a part of the overall guideline development process. These are aspects concerned with formulating a key question, systematically searching and gathering the related evidence, and assessing its potential for bias, level of precision, and other elements of quality. The quality of the evidence, in combination with a number of other factors, guides the strength of the recommendation a guideline panel should provide. Some evidence-grading systems can go beyond these aspects and provide guidance on steps that are part of the overall guideline development process, such as those relating to stakeholder representation, dissemination and communication, and conflicts of interest. It has previously been reported that a lack of standardization in grading evidence and guideline development methodology can lead to varying quality of guideline recommendations [10,11]. A systematic review on the guideline development process would be useful to the guideline development community to help improve transparency in the process.

Our aims were: first, to establish a comprehensive inventory of evidence-grading systems for medical tests. We wanted to compare the methods in each of these systems, with a focus on those aspects related to appraising evidence, linking different bodies of evidence, and translating evidence into recommendations; as a secondary aim, we compared the extent to which more general guideline development processes were included in these grading systems.

## Methods

### Definitions of a guideline development process and grading system

**Figure 1:**
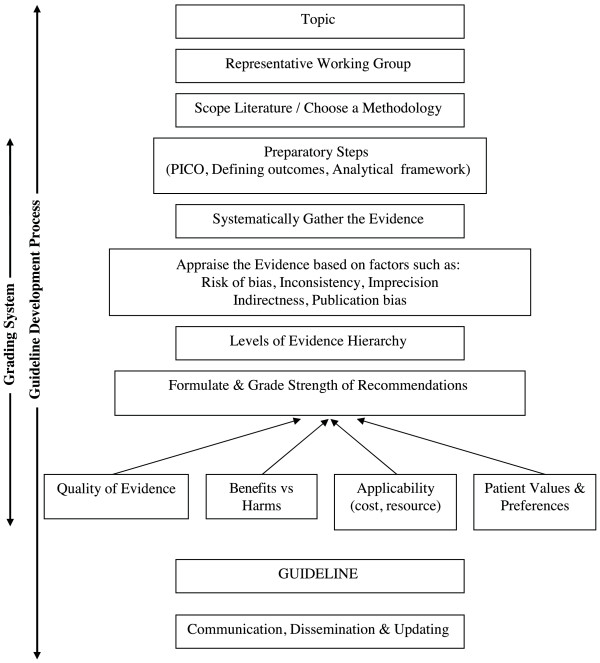
Main steps in guideline development.

For the purposes of this review, we defined an evidence-grading system to be a subset of the overall guideline development process. We defined a guideline development process to be one that begins by defining a topic of interest, then conducting a scoping of the literature in order to assess feasibility of the chosen topic. Once feasibility is ascertained, specific key questions that the guideline will address should be explicitly defined. This is followed by systematically gathering the available evidence and evaluating its quality; then formulating recommendations based on a number of factors such as the quality of the gathered evidence, applicability, resource issues, benefits versus harm, etc. We defined a grading system to be the subset of this series of processes: those that address aspects concerned with formulating a key question, systematically searching and gathering the related evidence, then assessing its quality and formulating recommendations (Figure 1).

### Identification of grading systems

Because grading systems are often described and used within the setting of a guideline development body, such as the National Institute for Health and Clinical Excellence (NICE), and are not always published in peer-reviewed journals, we felt it necessary to used a multi-staged approach that involved searching various information platforms up to March 2013 (Additional files 1 and 2).

To be included in our review, the description of a grading system must explicitly state that it can be used for grading evidence and making recommendations for medical tests. The system must include a ‘levels of evidence’ and ‘strength of recommendations’ table. Systems that were non-English or employed only a checklist or other quality assessment tool (*e.g.*, QUADAS) to assess the quality of the evidence and stopped there were excluded. Articles reporting a specific group process in developing a guideline, rather than a grading system that could be used as a tool for developing guidelines in general, were excluded.

We used a list of key articles as a starting point. The key article list contained a number of articles on available approaches to evaluating evidence and making recommendations in general and specific to medical tests. These articles were identified by the authors of this study. We looked at how these articles were indexed in Pubmed and analyzed frequency of the terms used to index these articles. From these, we picked a range of most frequently used terms, which we then combined with a selection of free text words relating to diagnostic test accuracy (Additional file 1). We selected these terms from existing test accuracy search filters [12,13] to reduce the number needed to screen and the associated potential for error.

We also searched the websites of various guideline developers (Additional file 2). These were selected based on citations from the key articles mentioned above and from the experiences and knowledge of the authors through informal discussions. The first author also screened documents on medical test guidelines retrieved via personal correspondence.

We hand searched references of the key articles as well as those articles that fitted the inclusion criteria that were retrieved from Pubmed to identify additional grading systems. The first author made a preliminary selection of articles by checking the titles and/or abstracts. A definite selection was made by the first author after reading the full text of the selected articles.

### Data extraction and presentation

We aimed to describe the identified grading systems in a consistent, standard manner using relevant categories. As a starting point, we referred to the categories in the Appraisal of Guidelines for Research and Evaluation (AGREE) instrument [14], a tool to assess the quality of clinical practice guidelines. We adapted the domains and items from the AGREE instrument via several rounds of informal discussions among the authors. From this, we defined two sets of assessment categories: methodological characteristics and process characteristics (Tables 1 and 2).

**Table 1:** Methodological characteristics

**No.**	**Category/sub category**	**Description**
**Methodological characteristics: features relating to how evidence is gathered, appraised and recommendations developed**		
**1**	**Structuring the search**	
1a-g	Preparatory steps prior to evidence collection	Preparatory steps are clearly outlined prior to beginning the literature search. Preparatory steps defined as any step that defines the remit of the guideline, such as scoping of the literature*, identify key question(s), define outcomes of importance, create a clinical scenario/care pathway and/or analytical framework **
**2**	**Searching for the evidence**	
2a	Explicit methodology exists	A systematic search strategy (*e.g.*, a systematic literature review) for gathering the evidence is described
2b	Minimum no. of databases	A minimum no. of databases is specified which need to be included in the search strategy
**3**	**Types of evidence gathered**	
3a-c	Accuracy data	The search for evidence extends beyond test accuracy to include other evidence such as patient important outcomes (*e.g.*, quality of life), cost and resource, legal and ethical issues etc.
	Patient important outcome data	
	Other	
**4**	**Appraising the evidence**	
4a	1st tier (individual study level)	Evidence is appraised at the individual study level
4b	2nd tier (as a body of evidence *e.g.*, systematic review)	Evidence is appraised as a total body (*i.e.*, systematic review)
4c	3rd tier (combining different bodies of evidence)	Different bodies of evidence are brought together and appraised (*i.e.*, combining evidence derived from different systematic reviews or other forms of evidence reports on cost, quality of life measures etc.)
**5**	**Explicit criteria for appraising the evidence**	
5a-c	1 tier (individual study)	Criteria used to appraise the evidence at each tier is explicit. For instance, is there a quality checklist used, what are the levels of evidence, is appraisal done in duplicate by different reviewers, is there an evidence table compiled, what other criteria are used to assess evidence quality
	2 tier (as a body of evidence *e.g.*, systematic review)	
	3 tier (combing different bodies of evidence)	
**6**	**Formulating recommendations**	
6a	Methods on how recommendations are derived	Explicit method(s) exist to formulate the recommendations and how final decisions are arrived at. Methods include for example, a voting system, formal consensus techniques (*e.g.*, Delphi, Glaser techniques). Areas of disagreement and methods of resolving them should be specified
6b	Guidance on wording of recommendations	Guidance is provided on how recommendations should be worded to provide clear, unambiguous recommendations
6c	Patient important outcomes considered	Patient important outcomes are explicitly considered in the recommendation formulation stage
6d	A method exists to translate indirect evidence into recommendations	An explicit methodology exists on how indirect evidence (*i.e.*, accuracy data) is translated into recommendations
6e	Applicability of recommendations considered	Potential organizational barriers and cost implications of recommendations are considered. For instance, applying the recommendations may require changes in the current organization of care within a service or a clinic which may be a barrier to using them in daily practice. Recommendations may require additional resources in order to be applied. For example, there may be a need for specialized staff, new equipment, or an expensive drug treatment
**Total**	**6 categories/23 subcategories**	

*Scoping of the literature is defined as a very broad search of the literature relevant to the condition that is to be the topic of the guideline. No attempt is made to focus on specific questions at this stage. The intention is only to establish the general extent of the literature in the clinical area to see if there is likely to be sufficient good quality evidence to make evidence based guideline feasible.

**A clinical scenario is defined as a scenario that addresses the intended use of test including setting *e.g.*, primary or specialist care; how test will be applied *e.g.*, screening, diagnosis; who will be tested *e.g.*, general population or high risk groups.

**Care pathway is defined as the diagnostic sequences, treatments, monitoring, retreatment, treatment for side effects and complications that are part of the test-treatment pathway. A flow chart or other diagram can be used to illustrate the pathway.

**Analytical framework is defined as an overarching framework showing linkages of the clinical scenario, the intermediate and health outcomes of interest, and the key questions to be addressed.

**Table 2:** Process characteristics

**No.**	**Category/sub category**	**Definition**
***Process characteristics: basic guideline requirements relating to the guideline development process***		
**1**	**Scope & purpose**	
1a	Overall aim	The overall aim of system is explicit. The system is specific to medical tests
**2**	**Stakeholders**	
2a	Relevant professional groups	The system requires involvement of relevant professional groups
2b	Piloted among users	The system has been piloted among guideline developers and reports on user feedback
**3**	**Clarity & presentation**	
3a	Clear organization and content layout	The system contains a chronological description of the process for developing guidelines.
3b	Recommendations easily identifiable	The system promotes a clear and structured layout for presenting recommendations *e.g.*, summarized in a box, typed in bold, underlined or presented as flow charts or algorithms
3c	Examples provided	The system provides adequate examples in the form of, tables, forms, layouts of evidence summaries *e.g.*, in GRADE known as ‘’evidence profile’
3d	Glossary	The system includes a glossary explaining terminology
**4**	**Dissemination & communication**	
4a	External peer review recommended	The system recommends external peer review of completed guidelines
4b	Recommends methods for dissemination	The system recommends methods for dissemination and communicated of completed guidelines to target audience(s)
4c	Procedure for updating guideline	The system provides an explicit procedure for updating the guideline
**5**	**Editorial independence**	
5a	Addresses conflicts of interest	The system specifies the need to address conflicts of interest of guideline members and information on funding
5b	Addresses funders	
**Total**	**5 categories/12 subcategories**	

Methodological characteristics are focused on the grading system within a guideline development process. These were defined based on how explicit a grading system was in describing how the evidence is gathered, its quality assessed and recommendations derived. Here, we defined six categories and a total of 23 subcategories (Table 1). Process characteristics are basic guideline requirements relating to the more general guideline development process (Figure 1), such as the need to include relevant experts in the guideline panel, providing clear and structured guidance on the process to follow when making a guideline, on the need for guideline panels to address editorial independence, and other process features. To reflect these, we defined in total five categories and 12 subcategories (Table 2).

We tested the applicability of each category and subcategory by extracting relevant information from a random selection of grading systems. Data extraction was done in duplicate and differences resolved through discussion between two reviewers. A third reviewer was involved when consensus could not be reached. A review protocol detailing the above methods was created by the first author (Additional file 3). Since there was no research intervention, our study was exempt from review by an ethics committee according to the Dutch Law.

## Results

**Figure 2:**
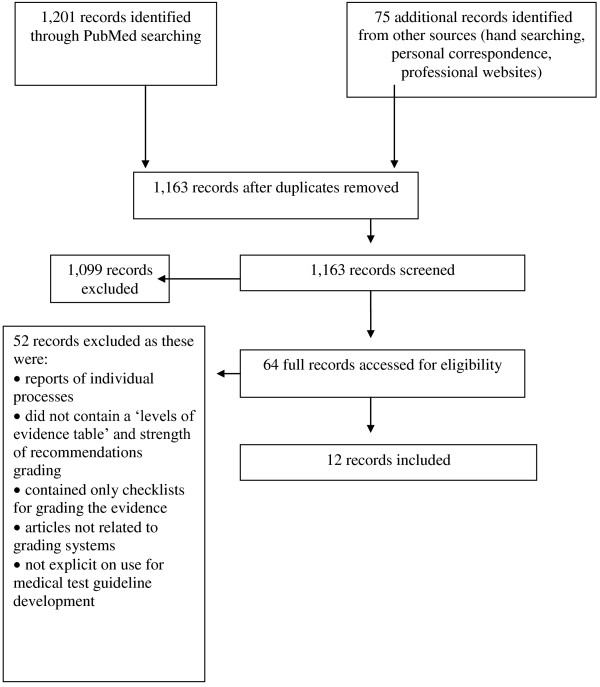
Summary of search strategy.

A total of 1,163 records were screened by title and/or abstract resulting in 64 full text records being retrieved (Figure 2). Twelve grading systems [15-31] were finally identified for inclusion, two of which—Evaluation of Genomic Applications in Practice and Prevention initiative (EGAPP) and NICE’s Diagnostic Assessment Programme (DAP)—were specific to medical tests [22,26]. We included the NICE DAP as at the time of this review it was unclear if NICE would also adopted the Grading of Recommendations Assessment, Development and Evaluation (GRADE) methodology for its diagnostic guidelines [32].

### Methodological characteristics

### Structuring the search

**Figure 3:**
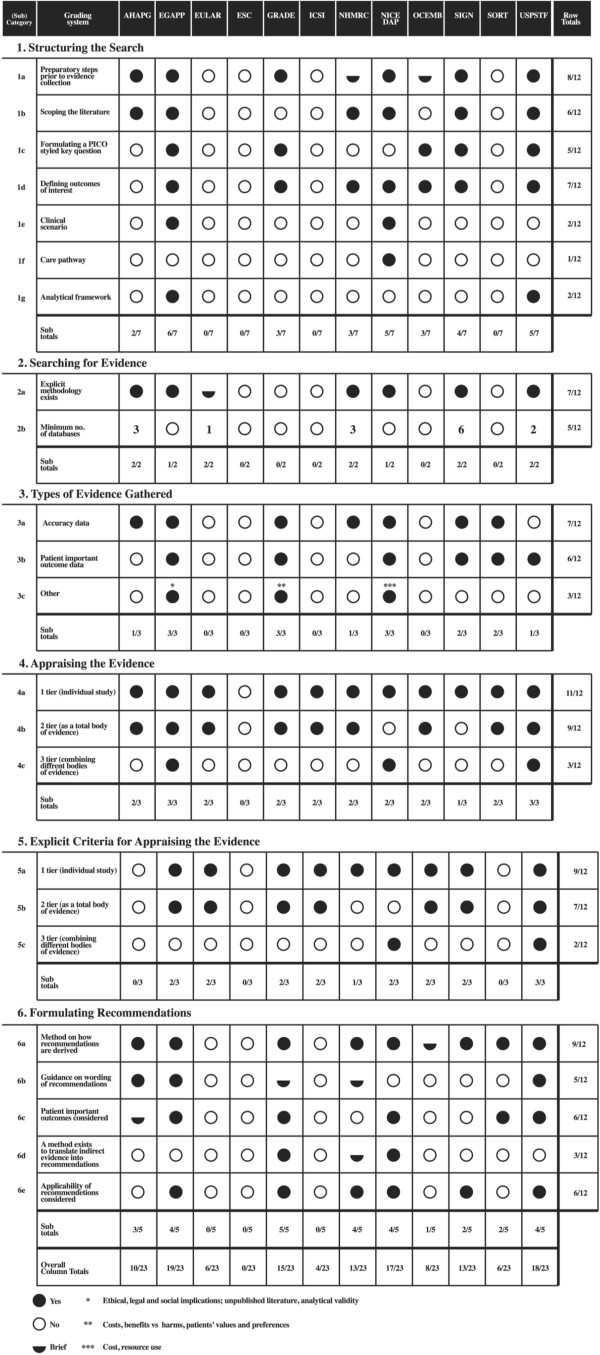
Methodological characteristics.

EGAPP was the most comprehensive for this category, covering six out of the seven subcategories, followed by NICE DAP and the U.S. Preventive Services Task Force (USPSTF), which each addressed five out of the seven subcategories listed (Figure 3). The subcategories ‘Preparatory steps prior to evidence collection’ (8/12), ‘Defining outcomes of interest’ (7/12), ‘Scoping the literature’ (6/12), ‘Formulating a Patient Intervention Comparison Outcome (PICO) styled key questions’ (5/12) were the most commonly covered features in this category across all the grading systems (Figure 3). The subcategory ‘Clinical scenario’ (EGAPP and NICE DAP) and ‘Analytical framework’ (EGAPP and USPSTF) featured only in two grading systems (Figure 3). The NICE DAP was the only system that included a feature called the ‘care pathway,’ defined as a pathway that shows the entire sequence of tests and treatments related to the test being evaluated.

### Searching for the evidence

Having an explicit search methodology as part of evidence gathering was addressed in seven out of 12 systems, to varying degrees of detail. Less than half of the grading systems were explicit on the number and type of databases the search should include (5/12) (Figure 3).

### Types of evidence

The European League Against Rheumatism (EULAR), European Society of Cardiology (ESC), Institute for Clinical Systems Improvement (ICSI) and the Oxford Centre for Evidence-based Medicine (OCEMB) were not explicit on the types of evidence that needed to be collected. Half of the systems (6/12) specified the need to collect patient important outcome data of which only three (EGAPP, GRADE and NICE DAP) explicitly required other types of evidence to be gathered such as costs, quality of life, contextual implications of the test such as ethical, legal, and social implications and information on resource use.

### Appraising the evidence

All systems except one (ESC) required users to grade the quality of the evidence at the individual study level, nine of which outlined specific criteria to be used. Nine systems also explicitly required the evidence to be graded collectively as a ‘body of evidence,’ and seven provided specific criteria by which this could be done. Only NICE DAP and the USPSTF provided explicit guidance on appraising different bodies of evidence. The EGAPP acknowledges in its grading system the need to link and appraise different types of evidence in the appraisal and guideline development for medical tests, given that accuracy alone is indirect evidence on patient important outcomes. However, it does not provide an explicit method by which this can be done.

### Formulating recommendations

Methods for making recommendations were explicitly provided in most systems (9/12). Only six systems reported the inclusion of patient important data when making recommendations. Explicit methods for translating accuracy evidence to recommendations were provided in only three of the 12 systems (GRADE, NICE DAP and the National Health and Medical Research Council (NHMRC)).

Current publications on applying GRADE to diagnostic tests describe the use of a considered judgment process where the guideline panel makes an informed judgment based on presumed patient important outcomes for patients with true and false positive test results, and true and false negative test results [17,18]. Other factors considered are quality of the evidence, benefits versus harms of the test, patient values and preferences, and cost [17]. In the NICE DAP manual, the guideline panel takes into account the evidence from three aspects when making recommendations: diagnostic accuracy evidence, clinical effectiveness and cost effectiveness [26] (Table 3). Cost-effectiveness of the diagnostic technology is the aspect most explicitly assessed through the use of models, although details of how the models are generated and how the related evidence is gathered are not described. The NICE DAP manual explicitly states that the extent to which the considerations described under diagnostic accuracy and clinical effectiveness are taken into account are a matter of the guideline panel’s discretion.

**Table 3:** Summary of main features in GRADE, NICE DAP and the NHMRC systems for moving from evidence to making recommendations

	**GRADE**	**NHMRC system**	**NICE DAP system**
**Evidence on accuracy**	Using a considered judgment process, derive presumed PIO for the four accuracy groups: TP, TN. FP, FN	**Evidence base aspects considered:**	**Diagnostic accuracy aspects considered:**
			- Validity
		- Number of studies	- Inclusiveness of underlying data
		- Level of evidence	- Meta analysis techniques
		- Risk of bias	- Cut off points
			- Uncertainty of data
**Factors considered when moving to recommendations**	Then consider:		-
	- Quality of evidence gathered	**Clinical impact:** not explicitly explained	**Clinical effectiveness:**
	- Patient’s values and preferences	**Generalisibility:** how well does the body of evidence match the the body of evidence match thepopulation and clinical setting being targeted by the guideline	- Nature and quality of evidence derived from expert,
	- Costs	**Applicability:** is the evidence body relevant to the Australian healthcare context and culture	- Lay members and stakeholder judgments
	- Benefits vs harms	**Evidence base:**	- Uncertainty of evidence and differences in evidence gathered under research conditions vs in actual
		- Number of studies	- Clinical practice
		- Level of evidence	- Greater benefits or harms in subgroups
		- Risk of bias	- Risks and /or benefits of technology from patients perspective
			- Position of technology in overall care pathway and available alternative treatments
		**Consistency:** not explicitly explained	**Cost Effectiveness:**
			- Impact on patient outcomes
			- Robustness and appropriateness of model
			- Plausibility of inputs and assumptions made in economic model
			- Evaluation of the modeling approach and related evidence effectiveness ratios (ICERs) generated by the models
			- Range and plausibility of the incremental cost-
			- Likelihood of decision error and consequences
			- Degree of clinical need of patients under consideration
			- Potential for long term benefits of innovation

The NHMRC’s process of moving from evidence to making recommendations is the least explicit, in comparison to the GRADE and NICE DAP processes. The guideline panel rates the evidence according to five factors: evidence base (as determined by the levels of evidence hierarchy with systematic reviews having the highest level), consistency, clinical impact, generalizability, and applicability (Table 3). With the exception of the evidence base, an explicit methodology as to how the panel should rate these factors is absent. It appears here again that a considered judgment process is utilized based on the discretion of the guideline panel.

### Process characteristics

**Figure 4:**
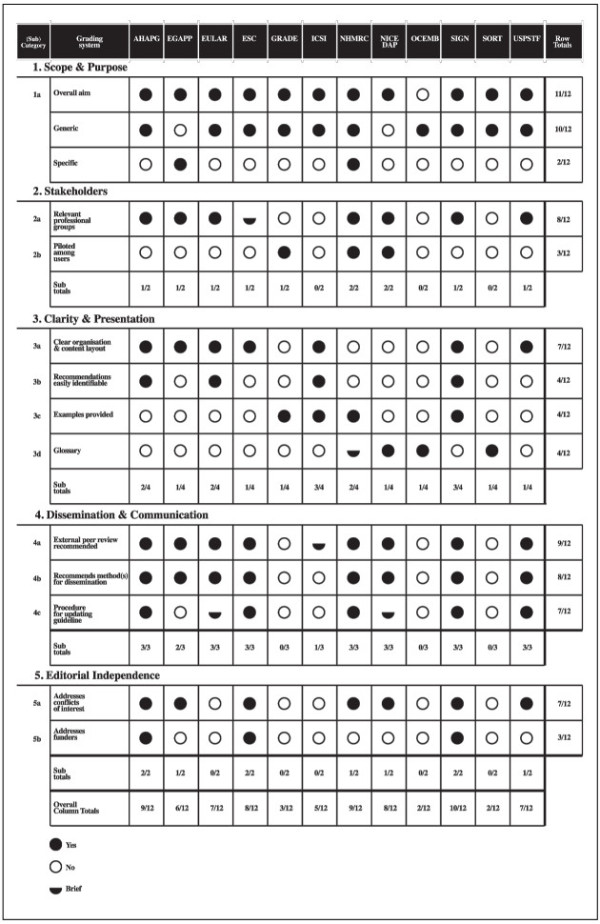
Process characteristics.

Characteristics most likely to be addressed in the 12 grading systems were the need for involvement of relevant professional groups during guideline development (8/12), the need for external peer review of completed guidelines (9/12), and the inclusion of recommendations on methods for dissemination of completed guidelines to target audiences (8/12) (Figure 4). The overall aim of 11 out of the 12 grading systems was explicitly stated.

Other characteristics commonly addressed included the presence of a clear content layout and organization (7/12), the presence of a procedure for updating guidelines (7/12), and the need for guideline developers to address conflicts of interest in a guideline (7/12).

Piloting of the grading system and information on funding were the least likely process characteristics reported, with only three of the 12 systems (Figure 4) fulfilling this category. Although we know (via personal correspondence) that the GRADE system has been piloted at a number of international meetings and forums, it was not explicitly reported in the publications assessed for this work [15-17]. Other less commonly addressed features include the provision of a glossary (4/12), key recommendations being easily identifiable in the guideline (4/12), and provision of example tables, forms, and other layouts to guide developers on the structure for developing a guideline (4/12) (Figure 4).

### Overall

**Figure 5:**
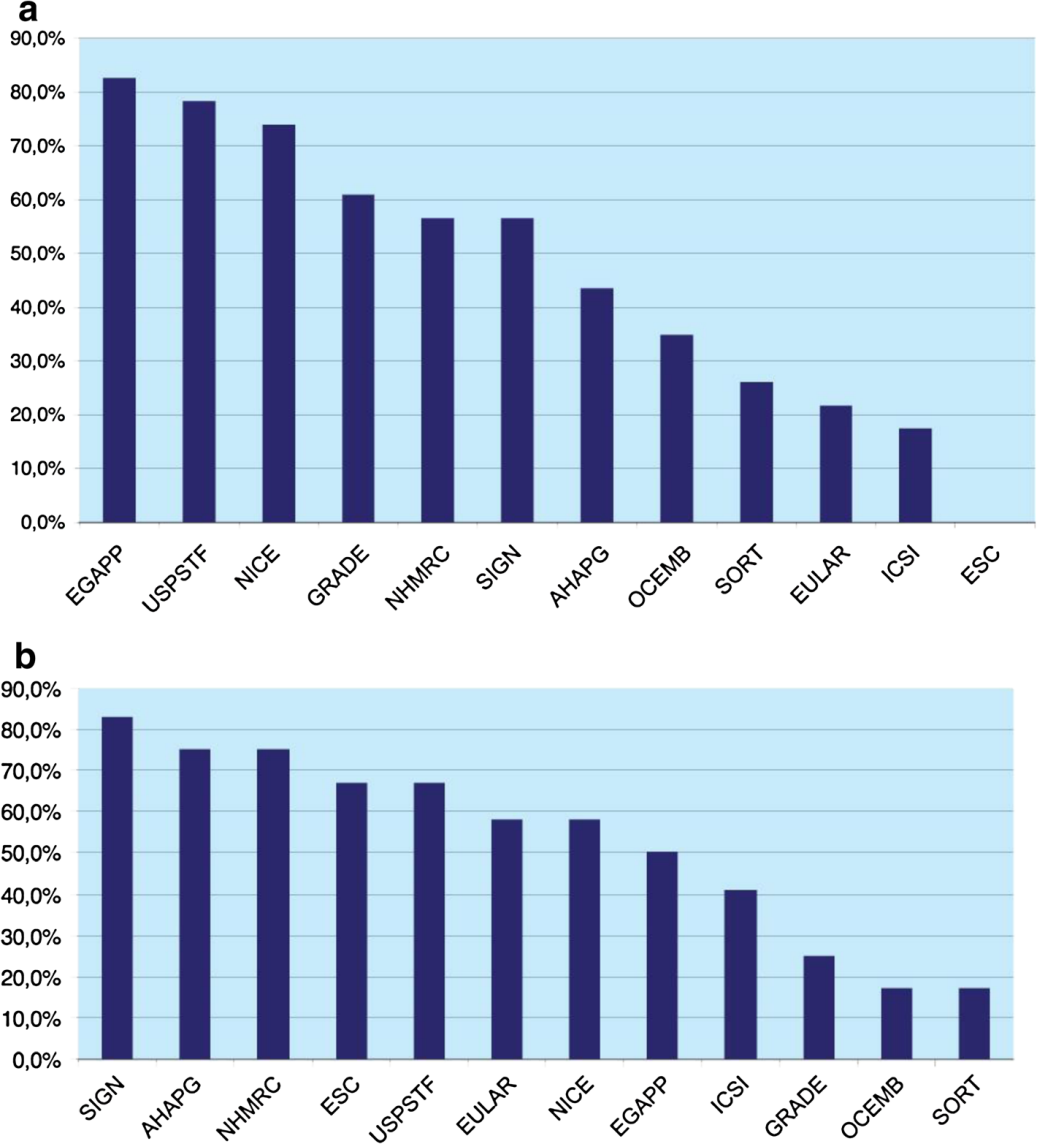
Overview: (a) methodological characteristics (b) process characteristics.

All 12 grading systems varied in the extent to which they addressed process and methodological characteristics. On the whole, we found a wide variation in terminologies used. Methodologically, out of the 23 subcategories defined, the EGAPP (19/23) followed by the USPSTF (18/23) and NICE (17/23) were the most comprehensive (Figure 3). The ESC was the least comprehensive where none of the 23 subcategories were met followed by ICSI (4/23), EULAR (6/23) and Strength of Recommendation Taxonomy (SORT) (6/23) (Figure 5a). The Scottish Intercollegiate Guidelines Network (SIGN), NHMRC and the American Heart Association Practice Guidelines (AHAPG) systems were the most comprehensive when it came to process features, with SIGN addressing 10 of the 12 process subcategories defined and AHAPG and NHMRC nine out of 12 each. OCEMB, SORT, GRADE and ICSI addressed the least number of process features (Figure 5b).

## Discussion

In this review, we identified 12 eligible evidence-grading systems that could be used by guideline developers to develop guidelines for medical tests. The EGAPP, USPSTF, NICE, GRADE, and NHMRC systems addressed, to differing degrees of explicitness, the need for and appraisal of different bodies of evidence, the linking of such evidence and its translation into recommendations. However, no one system adequately addressed the complexity of gathering, assessing, and linking different bodies of evidence, which is one of the challenges guideline developers of medical tests face. All 12 varied in basic guideline development features.

We have been able to include a number of grading systems available to guideline developers [1,33]. While we employed multifaceted search strategies in an attempt to be as comprehensive as possible, it is possible we have not identified all systems available. Similar difficulties of gathering grading systems have been reported previously [2,10]. Because we found no single repository that stores such systems, guideline developers looking for available grading systems or those interested in comparing such systems may face similar challenges. The wide variation in terminology usage across the systems is an issue raised previously by other researchers [10].

To describe the process characteristics, we felt adapting the domains from the AGREE instrument was the most appropriate, given that this is an instrument developed to assess the quality of clinical guidelines. However, AGREE was developed to assess the quality of the final product, whereas we looked at the guideline development process and thus we adapted the domains to fit this aim.

For the methodological characteristics relating to how evidence is gathered, assessed, and recommendations derived, the AGREE instrument does not address these aspects in a manner that was specific to the issues in medical test guideline development. The domain on ‘Rigor of Development’ contains eight items relating to how the evidence is gathered, assessed, and how recommendations are formulated [14]. While some of the items in the domain relate to how the evidence is appraised and whether an explicit methodology exists between the evidence and recommendations, we felt that one of the main issues in guideline development for tests—relating to the assessment of and linking of different bodies of evidence—is not addressed explicitly. Because there is currently no other instrument for appraising the quality of an evidence-grading system, or for defining the essential features it should contain [10], we had to define these categories based on the authors’ experiences and knowledge on the issues relating to challenges in medical test guideline development.

The objective of this review was not to make an analytical appraisal of the different grading systems available. To address that objective adequately is beyond the scope of this review. As such, the tables describing the systems in this review may not always appropriately discriminate the individual strengths and weaknesses of each system. It may also be worthwhile to note that not all of the systems included in this review may have been meant to be used independently, which may explain why they may have addressed process features poorly.

Two other prominent reviews of evidence-grading systems have been reported in the literature [1,2] A number of differences in scope and methodology exist between the review conducted by the Agency for Healthcare Research and Quality (AHRQ) [2] and that by the GRADE Working Group [1]. While the AHRQ review employed a systematic search strategy, it was not limited to identifying only systems applicable to medical tests. The review’s objective was to describe systems used to grade evidence. Hence checklists and such similar tools were included. The review was not concerned with the development of recommendations or the overall guideline development processes. The paper by Atkins *et al.*[1] did not employ a systematic search strategy. It was limited to six prominent rating systems known to the authors. Similar to the AHRQ review, it was focused on appraising each system on the aspect of rating evidence although the authors did extend this to include development of recommendations but stopped there. Neither review was limited to systems specific for medical tests.

Only three of the grading systems included in this review [15,23,26] had been user tested, which could explain why the other user test related features such as the availability of a glossary, template forms and tables, and key recommendations being easily identifiable were poorly addressed across the 12 systems. The extensive user testing done on the GRADE system for interventions and the incorporation of user feedback into its system could be one factor that has contributed to its popularity among guideline developers [33,34].

When it came to grading systems developed by specialty societies (AHAPG, ESC, EULAR, and SORT), these tended to be more comprehensive in addressing process characteristics related to guideline development, but were less thorough in covering features important for systematically gathering, assessing and making recommendations (Figure 3) (Figure 5a). For example, the AHAPG was the second most comprehensive system among the 12 grading systems, covering nine out of the 12 process characteristics (Figure 5b), but covered less than half of the methodological characteristics defined (10/23) (Figure 5a). Perhaps the starkest contrast was in the system by ESC where none of the 23 methodological categories were addressed. In comparison, the system fulfilled eight out of the 12 process subcategories, implying that grading systems developed by such organizations tended to be methodologically less comprehensive. This could explain the findings of Burgers *et al.* and Grilli *et al.* who report that guidelines produced by specialty societies tended to be of lower methodological quality compared to those produced by major guideline bodies [35,36].

Given the complexities in medical test guideline development, we defined a number of methodological characteristics as being particularly pertinent to medical tests and different to the methods for guideline development for interventions. The definition of clearly defined key questions is a first step in any guideline development. Because medical tests are often part of a test/treatment strategy, a PICO (Patient-Intervention-Comparison-Outcome) [37] styled key question is important but may not be adequate. In the NICE, EGAPP, and USPSTF systems, the PICO is supplemented with a broader framework. The clinical scenario, which both the EGAPP and NICE systems contain, addresses the intended use of the test, including the clinical setting (*e.g.*, primary care, specialty settings), how the test will be applied (*e.g.*, diagnosis or screening), and who will be tested (*e.g.*, general population or selected high risk individuals). Both the EGAPP and USPSTF go one step further to put the clinical scenario in question into the context of an analytical framework, which essentially is an overarching key question on clinical utility, *i.e.*, whether there is direct evidence that using the test leads to clinically meaningful improvement in outcomes.

While the NICE system does not contain an analytical framework, it defines a care pathway within which the clinical scenario is covered. The care pathway addresses a broader view on the test-treatment pathway that includes all aspects related to the application of the test and treatment sequences that may follow such as monitoring, retreatment, treatment for side effects and complications that may be experienced by the patient as a result of the test.

‘Which of the above preparatory steps is best?’ is a difficult question to answer. The NICE, EGAPP, and USPSTF obviously go beyond the typical PICO-styled key question definition, and would make for a more comprehensive assessment of a medical test than a grading system that does not include such components in its preparatory phase. The inclusion of features such as the clinical scenario, analytical framework, and/or care pathway helps to address the point reiterated many times that an evaluation of a test in context of its test-treatment pathway is more informative than the evaluation of its accuracy alone [9,38,39].

Rating the quality of evidence is another aspect in grading systems that is challenging for medical tests compared to interventions. Because of the indirectness of evidence about tests relative to patient important outcomes, different bodies of evidence may need to be gathered and assessed that are relevant to the patient outcomes being considered by the guideline panel. Often these data are either lacking or when available maybe in the form of observational studies that, in the level of hierarchy of studies, is classified as low quality [17].

Grading systems therefore need to provide explicit guidance to guideline panels on the types of evidence that need to be gathered in the absence of evidence of the effects on patient outcomes. Guidance is also needed on explicit methods on how these data can be assessed and linked to other bodies of evidence. Less than one-half of the grading systems in this review were explicit on the need for patient important data. Even fewer (three systems) mentioned the need to gather other types of data such as costs and resource utilization. EGAPP and NICE systems, both specific for medical tests, were the only two systems that were explicit on the need to gather such data, although they do not provide clear criteria on how such data should be evaluated. While we did not find this to be explicit in the current publications on GRADE for diagnostics [15-18], we are aware that this is an area currently being addressed by the GRADE Working Group (via personal correspondence).

A considered judgment process was the most commonly used method to move from evidence to making recommendations. GRADE, NHMRC, and NICE were the only three systems that provided clear criteria that should be considered when making this transition. Although GRADE’s process was the simplest and clearest amongst the three, none of the three systems involved an explicit method by which the different bodies of evidence could be translated into recommendations. NICE’s economic modeling, which formed one aspect of the process of moving from evidence to recommendations, was the only aspect that involved an explicit, structured methodology with clear cut-off points and guidance on the different recommendations to make based on the model thresholds [26].

## Conclusion

Clear guidance for evaluating, linking, and making recommendations based on different bodies of evidence beyond test accuracy is lacking in grading systems available today. The EGAPP, USPSTF, NICE, GRADE, and NHMRC systems address these aspects to varying degrees of comprehensiveness. There is a clear need for closer analysis of these features in each of these systems and for more targeted user testing among medical test guideline developers.

Existing grading systems for medical tests vary considerably in the extent to which they cover basic guideline quality requirements. The guideline development community can benefit from consensus and standardization regarding the basic features a grading system should cover. This could help to achieve consistency, improve transparency and, ultimately lead to the production of better quality guidelines.

## Abbreviations

AGREE: Appraisal of guidelines for research and evaluation; AHAPG: American heart association practice guidelines; EGAPP: Evaluation of genomic applications in practice and prevention initiative; ESC: European society of cardiology; EULAR: European league against rheumatism; GRADE: Grading of recommendations assessment, development and evaluation; ICSI: Institute for clinical systems improvement; NHMRC: National health and medical research council; NICE: National institute for health and clinical excellence; NICE DAP: NICE Diagnostic assessment programme; OCEMB: Oxford centre for evidence-based medicine; PICO: Patient intervention comparison outcome; PIO: Patient important outcomes; QUADAS: Quality assessment of diagnostic accuracy studies; SIGN: Scottish intercollegiate guidelines network; SORT: Strength of recommendation taxonomy; USPSTF: U.S. Preventive services task force.

## Competing interests

The authors declare that they have no competing interests.

## Authors’ contributions

GG defined the search strategy, conducted the literature search, data extraction and drafted the manuscript. MWL and MMGL carried out the data extraction each in duplicate with GG. PMMB initiated the original research idea. MWL, MMGL and PMMB participated in the definition of the data tables and figures. All authors contributed to and approved the final manuscript.

## Supplementary Material

Additional file 1 Full electronic search strategy used in Pubmed. Click here for file

Additional file 2 List of websites searched. Click here for file

Additional file 3 Review protocol. Click here for file
