# Supplementary material for: Guidelines for guideline developers: a systematic review of grading systems for medical tests
Source: Implement Sci. 2013 Jul 10;8:78. doi: 10.1186/1748-5908-8-78 (PMC3716938; doi:10.1186/1748-5908-8-78)
Supplement: Additional file 1 — Full electronic search strategy used in Pubmed. [file 1748-5908-8-78-S1.doc]

**Full electronic search strategy used in Pubmed until March 2013.**

(level of evidence[tiab] OR (strength[All Fields] AND recommendations[All Fields]) OR practice guidelines[tiab]) AND ("sensitivity AND specificity"[mesh] OR "specificit*"[tw] OR "false negative"[tw] OR "accuracy"[tw])
