# Supplementary material for: Guidelines for guideline developers: a systematic review of grading systems for medical tests
Source: Implement Sci. 2013 Jul 10;8:78. doi: 10.1186/1748-5908-8-78 (PMC3716938; doi:10.1186/1748-5908-8-78)
Supplement: Additional file 2 — List of websites searched. [file 1748-5908-8-78-S2.pdf]

List of websites searched

http://www.chestnet.org/accp/guidelines/development-process?page=0,6

http://www.sign.ac.uk/methodology/index.html

http://www.nhmrc.gov.au/guidelines/how-nhmrc-develops-its-guidelines

http://www.guidelines.gov/content.aspx?id=11881

http://www.cebm.net/index.aspx?o=1025

http://www.egappreviews.org/workingrp/methods.htm

http://www.gradeworkinggroup.org/publications/index.htm

http://www.icsi.org/guidelines_and_more/evidence_grading_system_6/

http://www.uspreventiveservicestaskforce.org/

http://www.nice.org.uk/

http://canadiantaskforce.ca/guidelines/

http://www.ahrq.gov/

http://www.eular.org/index.cfm?framePage=/recommendations.cfm

http://www.escardio.org/guidelines-surveys/esc-guidelines/about/Pages/rules-writing.aspx

http://www.openclinical.org/prj_agree.html

http://www.equator-network.org/research-projects/

http://www.thoracic.org/statements/
