# Supplementary material for: Guidelines for guideline developers: a systematic review of grading systems for medical tests
Source: Implement Sci. 2013 Jul 10;8:78. doi: 10.1186/1748-5908-8-78 (PMC3716938; doi:10.1186/1748-5908-8-78)
Supplement: Additional file 3 — Review protocol. [file 1748-5908-8-78-S3.doc]

**REVIEW PROTOCOL**

Our aim is to identify grading systems for medical tests that currently exist up til March 2013. In order to accomplish this, we feel it would be best to approach this via a systematic, multi pronged approach that involved searching various information platforms (Figure).

We will look at the indexing terms in Pubmed for a list of key articles and analyzed frequency of the terms used to index these key articles. The key article list contains a number of articles on grading the quality of evidence and making recommendations in general and specific to medical tests. These articles were identified either as a result of background reading by the first author or referred to the first author as key articles on the topic by co-authors. From these, we will pick a range of most frequently used terms to be used in the search strategy and combine these with relevant free text words selected from test accuracy search filters (DeVille 2000, Leeflang 2006) to narrow the search to articles focused on medical tests.

As grading systems are often described and used within the setting of a professional body responsible for guideline development such as the National Institute for Health and Clinical Excellence (NICE), and are not always published in peer-reviewed journals, we will therefore expand our search to include the websites of various professional guideline developers. These will be selected based on citations from the key articles mentioned above and from the experiences and knowledge of the authors through informal discussions. We will also screen any relevant documents on guideline development of medical tests obtained via personal correspondences.

We will hand search references of the key articles as well as those articles that fit the inclusion and exclusion criteria from the Pubmed search to identify any new grading systems not already identified.

*Inclusion/Exclusion criteria*

A priori inclusion/exclusion criteria will be used to define whether identified literature is relevant to the review. These criteria will be applied twice first at the title, or title and abstract review stage, and a second time at the article review stage. Studies that fail to meet the inclusion criteria once the full version is checked will be excluded; those that meet the criteria will be assessed. The inclusion/exclusion of studies will be done by the first author.

Inclusion criteria

- - Must explicitly state that it can be used for grading evidence in medical tests
  - Must include a structured format for grading evidence and making recommendations

Exclusion criteria

- - Non English
  - Systems that were only descriptive without a levels of evidence grading or strength of recommendations table
  - Systems that employed only a checklist or other quality assessment tool (e.g. QUADAS) to assess quality of study and stopped there

The characteristics for defining the grading systems will initially be adapted from the AGREE instrument. The AGREE instrument was created primarily as a measurement tool for assessing the quality of guidelines. As such, its primary target audience is guideline users principally clinicians and policy makers. In contrast, the primary aim of our table (and paper) is to provide an overview of the different grading systems available for medical tests. We expect guideline developers/panels looking for available methods to develop guidelines in this area to be our primary audience. With this in mind, we will adapt the domains and items from the AGREE instrument via several rounds of informal discussions among the authors to represent process and methodological features of grading systems. The predefined categories will be tested by describing a few included grading systems. Those characteristics for which valid information cannot be obtained will be discarded. These predefined categories will also be used to create the data extraction tables.

2 reviewers will independently extract the information from the selected grading systems into the data extraction tables. Any differences will be resolved through discussion.

Key articles list

Hand searching of references, personal correspondence

Internet search

Analysis of most frequently used indexing terms

Search strategy for Pubmed

Initial screening based on title/abstract

Final screening based on inclusion / exclusion criteria

Identified grading systems

Figure summarising the search strategy used to identify grading systems for medical tests

**References**

Devillé WL, Bezemer PD, Bouter LM. Publications on diagnostic test evaluation in

family medicine journals: an optimal search strategy. J Clin Epidemiol. 2000,

53(1):65-9.

Leeflang MM, Scholten RJ, Rutjes AW, Reitsma JB, Bossuyt PM. J Clin Epidemiol. 2006, 59(3):234-40.
